# Sensorimotor impairments during spaceflight: Trigger mechanisms and haptic assistance

**DOI:** 10.3389/fnrgo.2022.959894

**Published:** 2022-08-11

**Authors:** Bernhard Weber, Martin Stelzer

**Affiliations:** Institute of Robotics and Mechatronics, German Aerospace Center, Oberpfaffenhofen, Germany

**Keywords:** microgravity (μg), sensorimotor performance, cognitive load, haptic devices, force feedback (FF)

## Abstract

In a few years, manned space missions are planned in which the sensorimotor performance of humans will be of outstanding importance. However, research has repeatedly shown that human sensorimotor function can be impaired under conditions of microgravity. One way to compensate for these impairments is haptic feedback provided by the human-machine interface. In the current series of studies, sensorimotor performance was measured in basic aiming and tracking tasks. These tasks had to be performed using a force feedback joystick with different haptic settings (three spring stiffnesses, two dampings, two virtual masses, and no haptics). In two terrestrial studies, we investigated (1) the effects of cognitive load on performance in a dual-task paradigm (*N* = 10) and (2) which learning effects can be expected in these tasks in a longitudinal study design (*N* = 20). In the subsequent space study (*N* = 3 astronauts), the influence of microgravity and haptic settings of the joystick were investigated. For this purpose, three mission sessions after 2, 4, and 6 weeks on board the International Space Station (ISS), as well as terrestrial pre- and post-flight sessions, were conducted. The results of the studies indicated that (1) additional cognitive load led to longer reaction times during aiming and increased tracking error while aiming precision was not affected. (2) Significant learning effects were evident for most measures in the study on time effects. (3) Contrary to the expected learning trend, microgravity impaired the aiming precision performance of all astronauts in the initial phase of adaptation (2 weeks in space). No other significant effects were found. Intriguingly, these performance decrements could be compensated for with low to medium spring stiffness and virtual mass. The general result pattern provides further evidence that distorted proprioception during early adaptation to microgravity conditions is one main mechanism underlying sensorimotor impairment.

## 1. Introduction

As the next scientific milestone of space travel, space agencies are planning manned exploration missions to establish habitats first on the moon and eventually on Mars. In a first step, telerobotic systems will be used, in which astronauts remotely control robots on the celestial bodies from the safe environment of an orbital spacecraft. Thus, mobile robots can be navigated, and their robotic manipulators can be used to interact with the remote environment (e.g., Seedhouse and Llanos, 2021; Panzirsch et al., 2022). Apart from the huge technical challenges, the successful implementation of such scenarios depends, in particular, on the astronaut's sensorimotor skills. However, it is precisely these skills that can be impaired during spaceflight.

### 1.1. Sensorimotor impairments during spaceflight and explanatory approaches

Numerous empirical studies reported that the human sensorimotor system is affected by microgravity conditions and that, e.g., the performance of elementary motor tasks (e.g., aiming, tracking motions) is often impaired (e.g., Lackner and DiZio, 2000; Manzey, 2017). The extent of these sensorimotor impairments, however, depends on a variety of factors such as specific task demands, the context of exposure to microgravity (e.g., parabolic vs. space flight), and individual adaptability (refer to Bock, 1998; White et al., 2020; Weber and Proske, 2022 for reviews). Results for aiming tasks, e.g., are not completely consistent with respect to the accuracy of the aiming motions under microgravity conditions (Kanas and Manzey, 2008). However, a general slowing of motions in microgravity compared to terrestrial conditions was frequently reported for a variety of aiming tasks (Berger et al., 1997; Newman and Lathan, 1999; Sangals et al., 1999; Bock et al., 2001; Crevecoeur et al., 2010; Weber et al., 2022). Also, the accuracy of smooth tracking motions has been reported to decrease in microgravity (Kanas and Manzey, 2008; Weber et al., 2021).

Prior research provided several explanatory approaches for these sensorimotor performance decrements under conditions of microgravity, such as altered motion control, attentional deficits, and distorted proprioception. For example, Berger et al. (1997) and Mechtcheriakov et al. (2002) argued that the slowing of aiming motions is a strategic decision to avoid very fast movements because the occurring reaction forces on the weightless body and limbs cannot be compensated sufficiently in microgravity conditions. Apart from this explanation, researchers also argued that general stressors of spaceflight (e.g., sleep deprivation, high workloads) might cause an attentional deficit which also affects sensorimotor performance (Manzey et al., 1993, 2000; Bock et al., 2003; Fowler et al., 2008). A third explanatory approach has been discussed, which assumes that distorted proprioception is mainly underlying sensorimotor impairments in microgravity and numerous studies provided evidence supporting this assumption (refer to Weber and Proske, 2022 for a recent review).

The relevance of these mechanisms is seemingly dependent on the specific task demands. Altered motion control, e.g., was mainly reported for rapid full-arm aiming tasks (e.g., Berger et al., 1997; Mechtcheriakov et al., 2002). Evidence for disturbed proprioception has been found for aiming and tracking tasks, e.g., performed with a position-control joystick (i.e., joystick deflection is transferred into positions; e.g., Weber et al., 2021, 2022), while attentional deficits have been mainly reported for cognitively more demanding joystick-based tracking tasks with velocity control (i.e., joystick deflections are transferred into velocities; e.g., Manzey et al., 2000; Bock et al., 2003).

Moreover, the temporal patterns of when these mechanisms mainly manifest themselves are different: While changed motion control is a general, time-stable response to microgravity, proprioceptive deficits mainly occur in the initial phase of adaptation to microgravity (Kanas and Manzey, 2008; Weber and Proske, 2022). Attentional deficits do not follow a specific temporal pattern, since they are dependent on the individual level of mission-related workloads (Manzey et al., 2000).

### 1.2. Improving sensorimotor performance in microgravity through haptic assistance

One promising way to maintain human sensorimotor function in microgravity is to use external counterforces acting on the human limbs. In their groundbreaking study, Bringoux et al. (2012) demonstrated that attaching an elastic band to the arm can simulate the gravitational force and, thus, aiming motion accuracy can be recovered even in microgravity. Furthermore, it has also been shown that sensorimotor performance can be maintained in microgravity with specific haptic settings of the human-machine interface (HMI). Specifically, it was reported that aiming precision was improved in microgravity when applying a low centering spring stiffness at a force feedback joystick compared to no haptic support (Weber et al., 2022). Also, it was documented that low damping and stiffness are effective to support tracking accuracy in microgravity (Weber et al., 2021). Results suggested that besides the purely mechanical stabilization of movement, proprioception may be improved by these subtle haptic cues, thus compensating for the negative effects of microgravity.

The evidence of the above studies on haptic support was related to position control, but the question remains how the haptic design of the HMI has to be adapted when not positions, but velocities are commanded via the HMI. The operational effort, e.g., should be higher with velocity control, since there is no 1-to-1 correspondence between input and output, but an integral function is interposed (Zhai, 1996). This makes control mentally more demanding, especially for tasks with high temporal and precision requirements (Schäffler, 2016). For the telerobotic space missions described above, both position control and velocity control will be relevant and haptic assistance for both control modes is required. Telerobotic manipulation tasks, e.g., are usually performed with position control, while for rover navigation velocity control is more appropriate.

### 1.3. Present series of studies

The current study investigates the impact of microgravity conditions on sensorimotor performance in joystick-controlled aiming and tracking tasks in different stages of a spaceflight mission. As an extension of previous studies, where the effects of microgravity were investigated using position control, findings for task performance with velocity control were explored utilizing the same experimental paradigm. Analogous to the earlier studies of the authors, the effects of haptic support provided by a joystick (i.e., motion damping, spring stiffness, and virtual mass) were analyzed. Three different experimental studies were conducted in this study: two terrestrial studies and the spaceflight study. The terrestrial studies were carried out for a more fine-grained analysis and interpretation of the results of the spaceflight study.

In the first terrestrial study, the effect of limited cognitive resources on aiming and tracking performance was explored in a dual-task paradigm. In prior research examining the effects of microgravity vs. impaired attention on sensorimotor function (e.g., Manzey et al., 2000), the influence of the two mechanisms has been shown to be reflected in different dimensions of performance. The current study was performed to identify the effects of impaired attention (induced by a secondary task) on performance measures. In a previous study (Weber et al., 2019, with position control), it has been shown, e.g., that additional cognitive load mainly affects feedforward-controlled aspects of aiming performance (i.e., reaction times, rapid motions), while feedback-controlled aspects (i.e., precise target matching) were not affected at all. This knowledge is crucial to better distinguish between microgravity-related vs. attentional deficit effects in the spaceflight study.

In the second terrestrial study, the general time effects and potential interactions with haptic support were investigated. The spaceflight experiment utilized a longitudinal, repeated measures design, where time effects (such as learning) play a significant role. These effects were investigated in a terrestrial experiment with the very same experimental design as the latter spaceflight study. Previous studies, e.g., revealed that there are significant learning effects for aiming and tracking precision (Weber et al., 2021, 2022).

Finally, in the third study, the effects of microgravity and haptic support were explored during a spaceflight mission conducted on board the International Space Station (ISS). The study encompassed a terrestrial preflight session, three mission sessions (2, 4, and 6 weeks in space), and a terrestrial postflight session. While the two terrestrial studies were exploratory in nature and hence no hypotheses were formulated, two assumptions were made for the spaceflight study.

As described above, numerous factors play a role in the extent of sensorimotor impairments in microgravity. Accordingly, the time course of adaptation of the sensorimotor function to microgravity is, e.g., dependent on the specific task characteristics (refer to White et al., 2020 for a review). In joystick-controlled aiming and tracking tasks, it has been shown that microgravity-induced performance degradation is greatest in the initial phase of exposure, followed by a rapid adaptation over time (e.g., Kanas and Manzey, 2008, for a review). Furthermore, it has been shown that the initial performance degradation is particularly evident in tasks requiring a high level of motor precision (Fisk et al., 1993; Weber and Proske, 2022; Weber et al., 2022). Consequently, it was hypothesized that:

*Hypothesis 1: Aiming and tracking precisions decrease in the early phase of exposition to microgravity compared to the terrestrial baseline performance*.

Since velocity control is generally more cognitively demanding than position control, it is expected that, in contrast to previous studies by the authors (Weber et al., 2021, 2022), sensorimotor performance decrements which can be attributed to attentional deficits may also occur at later times of exposition (Manzey et al., 2000). However, these should primarily affect feedforward-controlled performance dimensions (e.g., reaction times) rather than feedback-controlled dimensions.

Furthermore, as in the earlier studies on aiming (Weber et al., 2022) and tracking performance (Weber et al., 2021) with position control, it is expected that the sensorimotor impairments in the early phase of adaptation can be mitigated by haptic support in the case of velocity control. With such haptic support provided by the input device, no gravitational forces are simulated (as in Bringoux et al., 2012), but the mechanical properties of the device (such as spring stiffness, viscous damping, and virtual mass) are used to stabilize the required movements. At the same time, these mechanical parameters provide a haptic representation of relevant kinematic parameters. Spring stiffness, for example, stabilizes deflections of the input device and thus simultaneously provides information about the current position of the hand-arm system. Viscous damping facilitates smooth movements by filtering out movement irregularities (such as tremors) and at the same time provides a haptic representation of the velocity. The mass fulfills a similar function, as jerky movements are physically impeded. Here, accelerations are haptically augmented. In normogravity conditions, moderate stiffness and damping have been shown to have a positive effect on aiming precision (e.g., Mayer and Cox, 2003; Lange, 2014). Moderate to high damping and mass also improve tracking performance (e.g., Jones, 1993; Weber et al., 2021). As mentioned above, even low intensities of damping and stiffness have been shown to effectively reduce sensorimotor impairments in microgravity which have been explained by distorted proprioception (Weber et al., 2021, 2022). Two aspects were discussed as possible explanations for this effect: (1) the additional haptic representation of position and velocity compensates for the proprioceptive deficit and (2) the movement against the forces applied by the device leads to an increase in muscle tone, which is otherwise reduced in microgravity, and thus proprioceptive function can be (partially) restored. The fact that higher intensities of haptic support which are beneficial under normal gravity conditions are sometimes detrimental under conditions of microgravity has been explained by the fact that high counterforces are more difficult to compensate for in weightlessness conditions (e.g., Weber et al., 2020). Altogether, we assume similar effects for velocity control:

*Hypothesis 2: Moderate haptic support (i.e., low spring stiffness, motion damping) is effective to maintain sensorimotor performance in microgravity*.

As in previous studies, the effects of virtual mass will also be explored, although there has been no evidence that additional mass helps maintain sensorimotor performance in microgravity.

The main objectives of this study are (1) a better understanding of the when and why of sensorimotor impairments in microgravity and (2) the how of selecting optimal haptic settings of the human-machine interface to maintain sensorimotor performance in such non-nominal gravity conditions.

## 2. Study 1: Cognitive load and sensorimotor performance

### 2.1. Materials and methods

#### 2.1.1. Sample

Ten subjects [1 f, 9 m; all right-handers; *M* = 25.1 (SD = 3.0) yrs.] participated in this study after having signed an informed consent form.

#### 2.1.2. Apparatus

**Figure 1:**
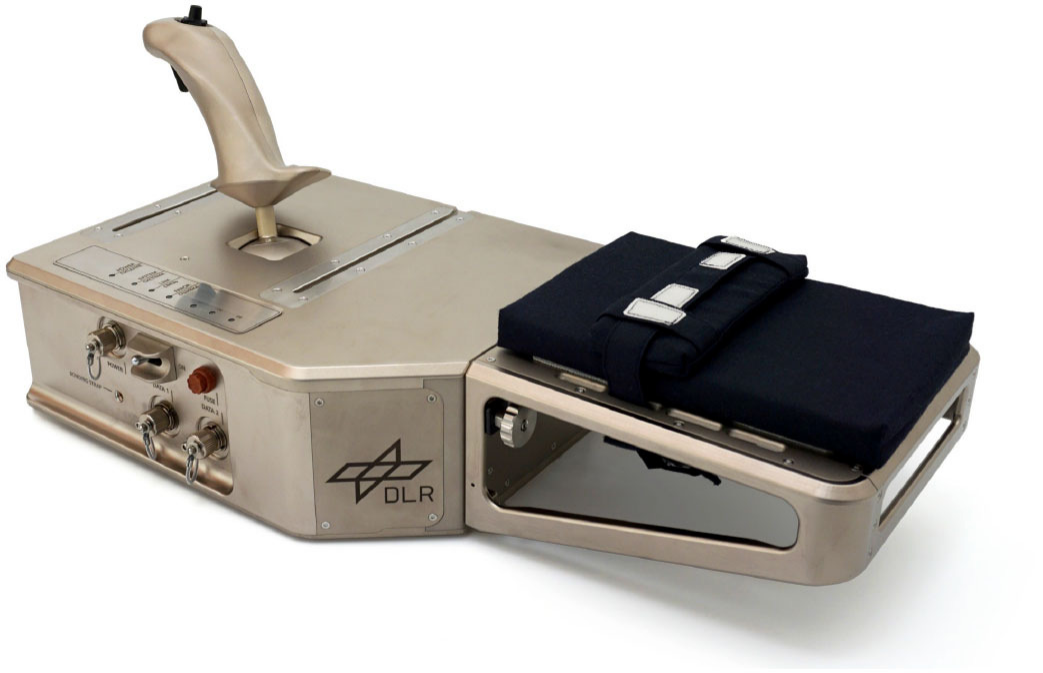
Force feedback joystick.

A force feedback joystick (Riecke et al., 2016) with a workspace of ±20° served as a human-machine interface (refer to Figure 1). A transfer function was implemented, where the movement velocity of the controlled cursor in the experimental simulation was proportional to the joystick's deflection (45 mm/s per degree). An armrest with an elbow strap ensured a comfortable and stable arm position, without restricting the required forearm motions in the experiment. The joystick was connected to the experimental notebook with a 15.4' display. There was a viewing distance of 70 cm from the subject's eyes to the display.

#### 2.1.3. Experimental tasks

##### 2.1.3.1. Aiming

**Figure 2:**
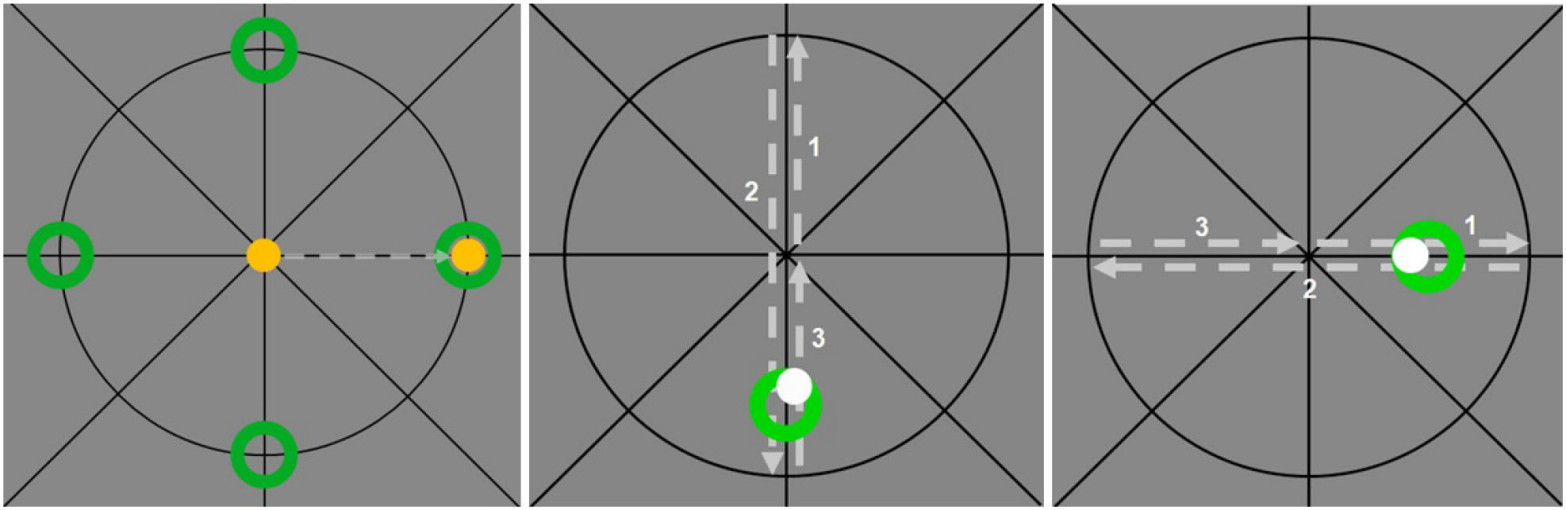
Experimental tasks. Aiming **(left)**: The four green target positions and the circular cursor (yellow) at the starting point and the target position. Tracking: The green target ring and the cursor (gray) during vertical tracking **(middle)** and horizontal tracking **(right)**.

A circular cursor in the graphical user interface (GUI) was controlled by the joystick. During the aiming task, subjects had to match four different target rings (upper, lower, left and right ring, refer to Figure 2, left) with this cursor. A target ring appeared at one of the pre-defined positions and the cursor (black color) had to be moved to the starting point in the center of the crosshairs. The cursor's color turned green to indicate that the starting point was reached and turned yellow after having held the position for 2 s. This color change indicated that the aiming task had to be performed immediately and subjects were instructed to match the target rings “as quickly as possible”. Upon reaching the target position in the inner of the ring (0.5 mm threshold) the cursor's color turned green and after holding this position for 0.5 s the cursor turned yellow and the task was successfully completed.

##### 2.1.3.2. Tracking

During tracking, the same starting point had to be matched and the cursor color switched to gray after having held the position for 2 s. Then the target ring immediately started moving along the vertical or horizontal axis of the crosshairs with a constant speed of 13 mm/s. Subjects were instructed to “match the target ring as precisely as possible” throughout the complete run. For both tracking directions, the target moved from the starting point to the two intersections of the axis and circle and returned to the start (refer to Figure 2, middle and right).

##### 2.1.3.3. Secondary task

In addition to the primary aiming and tracking tasks, there were conditions with an additional secondary task that had to be performed in parallel (dual-task condition). Specifically, a mental tracking task had to be performed (Wollesen et al., 2019) to induce additional cognitive load. Subjects had to count forward in 7s starting with 12 up to 103 and then backward again (12-19-26-33–103-96-89-82–12). A metronome tone prompted subjects to speak out the next number aloud at 4-s intervals. Thus, it was ensured that there was a continuous additional cognitive load in the dual-task condition.

#### 2.1.4. Experimental design and procedure

A 2 (Cognitive load) × 8 (Haptic condition) within-subject design was utilized. The order of the two Cognitive Load conditions, i.e., single-task (primary tasks only) and dual-task (primary and secondary task) was counterbalanced across subjects. Within both conditions, eight haptic conditions (refer to Table 1) had to be completed in random order. There was an isotonic reference condition (no haptics) and seven haptic conditions with three stiffnesses, two motion dampings, and two virtual masses. In all of the resulting 16 experimental conditions, the aiming task was completed first and the tracking task second. The experiment was conducted at the German Aerospace Center. Before starting the experiment, subjects were informed about the experimental tasks and procedure and signed an informed consent form. Subjects performed the experiment in a seated position. Seat height was individually adjusted. Each experimental condition started with a training trial with two randomly chosen targets for aiming and one randomly chosen tracking task (horizontal or vertical), to familiarize subjects with the next haptic setting. Subsequently, the main trials (four target positions and then two tracking directions, each in random order) were started.

**Table 1:** Haptic conditions.

**Experimental condition**	**Setting**
Isotonic	0.000 —
Spring stiffness 1	0.262 Nm/rad
Spring stiffness 2	0.524 Nm/rad
Spring stiffness 3	0.786 Nm/rad
Motion damping 1	0.15 Nm·s/rad
Motion damping 2	0.30 Nm·s/rad
Virtual mass 1	0.00187 kg·m^2^
Virtual mass 2	0.00374 kg·m^2^

#### 2.1.5. Data analysis

All data were recorded with a sampling rate of 100 Hz. For aiming, three temporal measures were calculated: (1) reaction time (RT = time from task start until the center of the cursor leaves the circular starting area with a radius of 3.25 mm around the crosshair center), (2) rapid motion time (RMT = time until the cursor touches the target ring—RT), and (3) fine motion time (FMT = time from touching the target ring until matching and holding the target position). For the tracking, a tracking error was calculated as the average Euclidean distance between the cursor's and the target's centers.

Repeated measures Cognitive Load * Haptic Condition * Direction ANOVAs (rmANOVA) were performed on all measures. In the case of non-sphericity, Greenhouse-Geisser corrections (GG) were made. α levels of post-hoc comparisons were adjusted using the Bonferroni method.

### 2.2. Results

#### 2.2.1. Reaction time

RmANOVA revealed a significant Cognitive Load main effect [*F*_(1, 9)_ = 34.42; *p* < 0.001, refer to Table 2], i.e., RTs were substantially longer in the dual-task condition (*M* = 0.976 s) compared to the single-task condition (*M* = 0.582 s). No other significant effects occurred.

**Table 2:** Effects of cognitive load on performance measures: Means, SD (in parentheses), and rmANOVA main effects.

	**No load**	**Load**	**ANOVA**
**Study 1: Cognitive Load Effects**			
RT [s]	0.582 (0.149)	0.976 (0.247)	*p* < 0.001
RMT [s]	1.647 (0.483)	1.783 (0.512)	*n.s*.
FMT [s]	4.209 (0.933)	4.494 (1.148)	*n.s*.
Tracking error [mm]	2.111 (0.660)	2.509 (0.720)	*p* < 0.10

#### 2.2.2. Rapid motion time

RmANOVA performed on the RMTs yielded a significant Haptic Setting main effect [*F*_(7, 63)_ = 10.39; *p* < 0.001, refer to Table 3]. *Post-hoc* comparisons, furthermore, revealed that Stiffness 3 led to significantly longer RMTs compared to the isotonic reference condition (*p* < 0.05). Additionally, a significant Direction main effect [*F*_(3, 27)_ = 3.82; *p* < 0.05] was found. Here, comparisons between the four directions revealed significantly longer RMTs for the upper target (*M* = 1.83 s) compared to the lower target (*M* = 1.66 s, *p* < 0.05).

**Table 3:** Effects of haptic settings on performance measures: Means, SD (in parentheses), and rmANOVA main effects.

	**Isoton**.	**Stiff. 1**	**Stiff. 2**	**Stiff. 3**	**Damp. 1**	**Damp. 2**	**Mass 1**	**Mass 2**	**ANOVA**
**Study 1: Haptic Setting Effects**									
RT [s]	0.848 (0.283)	0.788 (0.161)	0.816 (0.301)	0.774 (0.174)	0.784 (0.269)	0.809 (0.197)	0.728 (0.223)	0.686 (0.153)	n.s.
RMT [s]	1.527 (0.380)	1.966 (0.506)	1.924 (0.609)	1.959^*^ (0.535)	1.512 (0.374)	1.645 (0.399)	1.540 (0.370)	1.645 (0.427)	*p* < 0.001
FMT [s]	4.649 (1.424)	4.487 (0.968)	4.095 (0.784)	3.823 (0.812)	4.572 (0.978)	4.862 (2.037)	4.149 (0.888)	4.175 (0.693)	n.s.
Tracking error [mm]	2.109 (0.658)	2.754 (0.942)	2.263 (0.592)	2.104 (0.652)	2.564 (0.767)	2.561 (0.735)	1.956 (0.431)	2.169 (0.642)	*p* < 0.01

*Asterisks indicate significant differences compared to the isotonic condition and the corresponding means are in bold;*

* *p < 0.05*.

#### 2.2.3. Fine motion time

No significant effects were found in rmANOVA on FMTs.

#### 2.2.4. Tracking error

A marginal Cognitive Load main effect was revealed by rmANOVA [*F*_(1, 9)_ = 3.39; *p* < 0.10, refer to Table 2], i.e., Tracking Errors were greater with load (*M*_Load_ = 2.51 mm) than without load (*M*_NoLoad_ = 2.11 mm). Moreover, a significant Haptic Setting main effect was found [*F*_(3.49, 31.43)_ = 5.93; *p* < 0.01, refer to Table 3], although *post-hoc* comparisons did not reveal significant differences between the isotonic conditions and the other haptic settings. Finally, a significant Direction main effect [*F*_(1, 9)_ = 6.51; *p* < 0.05] showed that tracking errors were significantly larger for the vertical compared to the horizontal tracking task.

### 2.3. Discussion

Terrestrial Study 1 was conducted to compare sensorimotor performance with and without a mental secondary task, which is important to identify performance dimensions that are particularly affected by limited cognitive resources. Indeed, data revealed that aiming reaction times substantially increased and tracking error tended to be higher in the dual-task condition. No effects of cognitive load were found for rapid and fine aiming motion times.

Comparing the current results obtained with velocity control with the results of a previous study utilizing position control in an otherwise identical experimental paradigm (Weber et al., 2019), shows that the result patterns are not completely similar for aiming. Reaction times were increased by cognitive load in both studies. Seemingly, the time for feedforward movement planning is prolonged due to reduced cognitive resources. While rapid motion times were increased by the cognitive load during position control, this was not the case during velocity control. During position control, motion speed was significantly reduced in the dual-task condition. During motion execution, the initial feedforward plan for the motion is usually adjusted throughout motion execution (e.g., Taylor and Thoroughman, 2007). The slowing of motion allows us to adequately correct this plan when cognitive resources are limited. During velocity control, the rapid motion times were three times longer compared to position control even without cognitive load. Since the operating effort during fast movements is significantly higher with velocity control (Zhai, 1996), a much slower speed was generally commanded. This could explain why cognitive load did not lead to an additional slowing. The findings for fine motion times again were in line with the study on position control and no effects of cognitive load were evident. Regarding the haptic settings, results showed that the highest stiffness (0.786 Nm/rad) led to significantly longer rapid motion times. Obviously, the strong counterforce at the joystick prevented higher motion speeds.

During tracking, a constant motion speed has to be matched. Once the desired speed has been precisely achieved, the corresponding joystick deflection can be held constantly. As soon as a deviation occurs, a new feedforward plan must be generated and modified based on the visual feedback. Here, we found a trend that tracking errors increase under additional cognitive load.

In sum, we found evidence suggesting that cognitive load mainly affects the feedforward planning of rapid motions during aiming (as reflected in longer reaction times) and of motion corrections during tracking (as reflected in greater tracking error). No interaction was found between cognitive load and the effects of haptic settings on sensorimotor performance.

## 3. Study 2: Time effects on sensorimotor performance

### 3.1. Materials and methods

#### 3.1.1. Sample

Twenty subjects [6 f, 14 m; *M* = 36.4 (11.0) years] participated and signed an informed consent form.

#### 3.1.2. Apparatus and experimental tasks

The experimental setup and experimental tasks (aiming, tracking) were the same as in Study 1. There was no secondary task in this study.

#### 3.1.3. Experimental design and procedure

The same haptics conditions as in Study 1 were completed in five subsequent experimental sessions, resulting in a 5 (Sessions) × 8 (Haptic condition) within-subject design. To align the study designs of Study 2 with Study 3 (with *N* = 3 subjects), the order of the three haptic categories (stiffness [s], damping [d], and virtual mass [m]) was systematically varied by utilizing a 3 × 3 Latin Square design. Hence, subjects were assigned to one of the three category orders (1: s,d,m 2: d,m,s 3: m,s,d). The individual intensities within each haptic category were completed in ascending order. The isotonic reference condition was always the fourth condition. Again, subjects performed the experiment in a seated position and the study was also conducted at the German Aerospace Center. Instructions were the same as in Study 1, however, they were repeated at the beginning of each experimental session. Apart from this, Study 2 followed the same procedure as Study 1.

#### 3.1.4. Experimental schedule

The chronology of the five experimental sessions of Study 2 was based on the schedule of the space study (Study 3). In Study 3, the pre-mission session (T1) was conducted 91 days before the mission launch. The mission session T2 after 14 days, T3 after 27 days, and T4 after 41 days on board the space station. After having completed the 173 days mission, cosmonauts participated in the post-mission session (T5) 15 days after their return to earth. In Study 2, T2 was accordingly scheduled for 105 (=91+14) days, T3 118 (=91+27) days, and T4 132 (=91+41) days after completion of the first session (T1). However, T5 had to be conducted 162 days (instead of 279 [=91+173+15] days) later, to keep the drop-out rate of subjects low.

#### 3.1.5. Data analysis

Session * Haptic Condition *Direction rmANOVA were performed on the same measures with the same GG. corrections and α level adjustments for *post-hoc* comparisons as in Study 1.

### 3.2. Results

#### 3.2.1. Reaction time

RmANOVA yielded a significant Session main effect [*F*_(4, 76)_ = 8.82; *p* < 0.001, refer to Table 4]. RTs showed a clear learning trend and decreased across the five sessions [*T1: M* = 0.543 s; *T2: M* = 0.502 s; *T3: M* = 0.497 s; *T4: M* = 0.455 s; *T5: M* = 0.451 s; *p*_(T1vs.T4;T5)_ < 0.01]. Moreover, a significant Haptic Setting main effect was found [*F*_(4.13, 78.37, *GG*.)_ = 2.64; *p* < 0.05, refer to Table 5]. *Post-hoc* comparisons between the isotonic reference condition and the other haptic settings did not reach significance.

**Table 4:** Session effects on performance measures: Means, SD (in parentheses), and rmANOVA main effects.

	**T1**	**T2**	**T3**	**T4**	**T5**	**ANOVA**
**Study 2: Session Effects**						
RT [s]	0.543 (0.132)	0.502 (0.145)	0.497 (0.163)	0.455^***^ (0.122)	0.451^**^ (0.119)	*p* < 0.001
RMT [s]	1.338 (0.348)	1.377 (0.410)	1.398 (0.408)	1.329 (0.381)	1.174 (0.336)	n.s.
FMT [s]	6.369 (1.143)	5.853 (1.221)	5.450^**^ (1.274)	5.058^***^ (1.096)	5.089^***^ (0.994)	*p* < 0.001
Tracking error [mm]	2.971 (0.808)	2.777 (1.158)	2.351^**^ (0.834)	2.165^***^ (0.742)	2.237^**^ (0.972)	*p* < 0.001

*Asterisks indicate significant differences compared to Session T1 and the corresponding means are in bold;*

** *p < 0.01;*

*** *p < 0.001*.

**Table 5:** Effects of haptic settings on performance measures: Means, SD (in parentheses), and rmANOVA main effects.

	**Isoton**.	**Stiff. 1**	**Stiff. 2**	**Stiff. 3**	**Damp. 1**	**Damp. 2**	**Mass 1**	**Mass 2**	**ANOVA**
**Study 2: Haptic Setting Effects**									
RT [s]	0.488 (0.133)	0.481 (0.138)	0.475 (0.126)	0.468 (0.131)	0.524 (0.172)	0.514 (0.132)	0.484 (0.136)	0.484 (0.118)	*p* < 0.05
RMT [s]	1.256 (0.398)	1.379 (0.370)	1.421^***^ (0.363)	1.484^***^ (0.390)	1.269 (0.330)	1.355 (0.336)	1.283 (0.395)	1.298 (0.355)	*p* < 0.001
FMT [s]	6.022 (1.249)	5.608 (1.350)	5.242^*^ (1.036)	4.890^***^ (0.828)	5.730 (1.320)	5.594 (1.070)	5.744 (1.158)	5.682 (1.145)	*p* < 0.001
Tracking error [mm]	2.506 (0.889)	2.391 (0.807)	2.264 (0.749)	2.221^*^ (0.893)	2.707 (0.988)	2.834 (0.992)	2.550 (0.799)	2.528 (0.853)	*p* < 0.001

*Asterisks indicate significant differences compared to the isotonic condition and the corresponding means are in bold;*

* *p <0.05;*

*** *p <0.001*.

#### 3.2.2. Rapid motion time

A significant Haptic Setting main effect was found in rmANOVA [*F*_(3.74, 71.06, *GG*.)_ = 6.98; *p* < 0.001, refer to Table 5]. *Post-hoc* comparisons revealed that RMTs for Stiffness 2 and 3 were significantly longer than for the isotonic condition (both *p*s <0.001). Furthermore, a Direction main effect occurred [*F*_(3, 57)_ = 3.07; *p* < 0.05]. RMTs for the left target were longest (*M* = 1.38 s) and significantly longer than for the upper target (*M* = 1.27 s, *p* < 0.01). Finally, rmANOVA yielded a significant Direction × Haptic Setting interaction effect [*F*_(21, 399)_ = 5.05; *p* < 0.001], indicating that the negative effect of higher stiffnesses was solely evident for the lateral aiming motions (left and right target).

#### 3.2.3. Fine motion time

First, a significant Session main effect [*F*_(4, 76)_ = 13.78; *p* < 0.001, refer to Table 4] in rmANOVA was found. Data showed a clear learning trend across sessions (*T1: M* = 6.37 s; *T2: M* = 5.85 s; *T3: M* = 5.45; *T4: M* = 5.06 s; *T5: M* = 5.09 s; *p*_(T1vs.T3;T4;T5)_ < 0.01) Second, analysis yielded a significant Haptic Setting main effect [*F*_(4.21, 79.99, *GG*.)_ = 5.71; *p* < 0.001, refer to Table 5]. *Post-hoc* comparisons showed that FMTs were significantly shorter compared to the isotonic condition when being supported by Stiffness 2 (*p* < 0.05, 1tt) and Stiffness 3 (*p* < 0.001). Third, rmANOVA showed a significant Direction main effect [*F*_(3, 57)_ = 3.49; *p* < 0.05], however, no *post-hoc* comparison reached significance.

#### 3.2.4. Tracking error

Again, a Session main effect [*F*_(2.62, 49.82, *GG*.)_ = 13.43; *p* < 0.001, refer to Table 4] was found. Across sessions, a clear learning trend was evident (*T1: M* = 2.97 mm; *T2: M* = 2.78 mm; *T3: M* = 2.35 mm; *T4: M* = 2.16 mm; *T5: M* = 2.24 mm; *p*_(T1vs.T3;T4;T5)_ < 0.01). A significant Haptic Setting main effect [*F*_(4.19, 79.60, *GG*.)_ = 9.62; *p* < 0.001, refer to Table 5] occurred and *post-hoc* comparisons showed that tracking error was significantly reduced when applying Stiffness 3 compared to the isotonic condition (*p* < 0.05, 1tt). Finally, a Direction × Haptic Setting interaction effect reached significance [*F*_(7, 133)_ = 5.62; *p* < 0.001], indicating that haptic setting effects were only evident for the horizontal tracking task. Here, both dampings and Stiffness 3 led to significantly better performance compared to the isotonic condition (all *p*s <0.05).

### 3.3. Discussion

The main objective of the second terrestrial study (Study 2) was the determination of potential time effects on aiming and tracking performance in the same longitudinal study design as it was utilized in the spaceflight study (Study 3). Indeed, significant learning effects were found. Regarding aiming reaction times (RT), fine motion times (FMT) as well as tracking error, improvements were evident across experimental sessions, while no such effect was found for rapid motion times (RMT). This general result pattern is consistent with previous findings for position control (Weber et al., 2021, 2022). Although subjects had to acquire a new visuomotor mapping of hand motions and visual feedback when commanding velocities, the converging evidence indicate that the underlying learning processes with position and velocity control were quite similar. The decreasing response times could be an indicator for improving feedforward motion planning, but the fact that this learning trend was evident independently of motion directions and haptic settings, however, suggests that this is less plausible to assume (Ishihara et al., 2006; Weber et al., 2020). Additionally, the targets were visible before the respective aiming task start. Seemingly, subjects simply learned to better focus on the color switch of the cursor, indicating task started. There was no learning trend for the rapid motion part of the aiming task, which is not surprising as such basic point-to-point motions are very well-practiced and do not improve significantly across repetitions (Wolpert and Flanagan, 2010). The subsequent fine motion task, however, requires intensive processing of sensory feedback as well as the highest motor precision and similar studies have shown significant learning effects for such task demands (Shmuelof et al., 2012).

Similar to Study 1, haptic setting effects were evident for RMT. Subjects required more time with higher stiffnesses (0.524 and 0.786 Nm/rad), although this effect solely occurred during lateral aiming motions. This effect is mainly due to the fact that aiming motions with a joystick can be performed faster in the transversal (left-right joystick motion) compared to the sagittal motion plane (back-forth joystick motion) (Weber et al., 2022), and consequently, resistive forces have a stronger impact on motion times in this plane. Additionally, positive effects of the same stiffnesses were found during precision aiming. FMTs were significantly reduced compared to the isotonic reference condition. The fact that higher counterforces have a negative effect on RMTs and a positive effect on FMTs can be explained by the different task requirements in the two sections of the target task. The counterforces naturally hinder a fast aiming movement with a larger movement amplitude, but effectively stabilize the fine movement corrections in the target area. Finally, horizontal tracking performance improved with the highest stiffness and both dampings, which is also consistent with earlier findings on tracking with position control (Weber et al., 2021). These haptic setting effects were evident across all sessions, and no significant interaction effects were found. These findings on temporal effects in the longitudinal design were considered in the following spaceflight study, following the same methodological approach.

## 4. Study 3: Microgravity effects on sensorimotor performance

### 4.1. Materials and methods

#### 4.1.1. Sample

Three male cosmonauts aged 42, 45, and 53 years, participated in the study after having signed an informed consent form. Two of the cosmonauts already had space flight experience.

#### 4.1.2. Apparatus and experimental tasks

**Figure 3:**
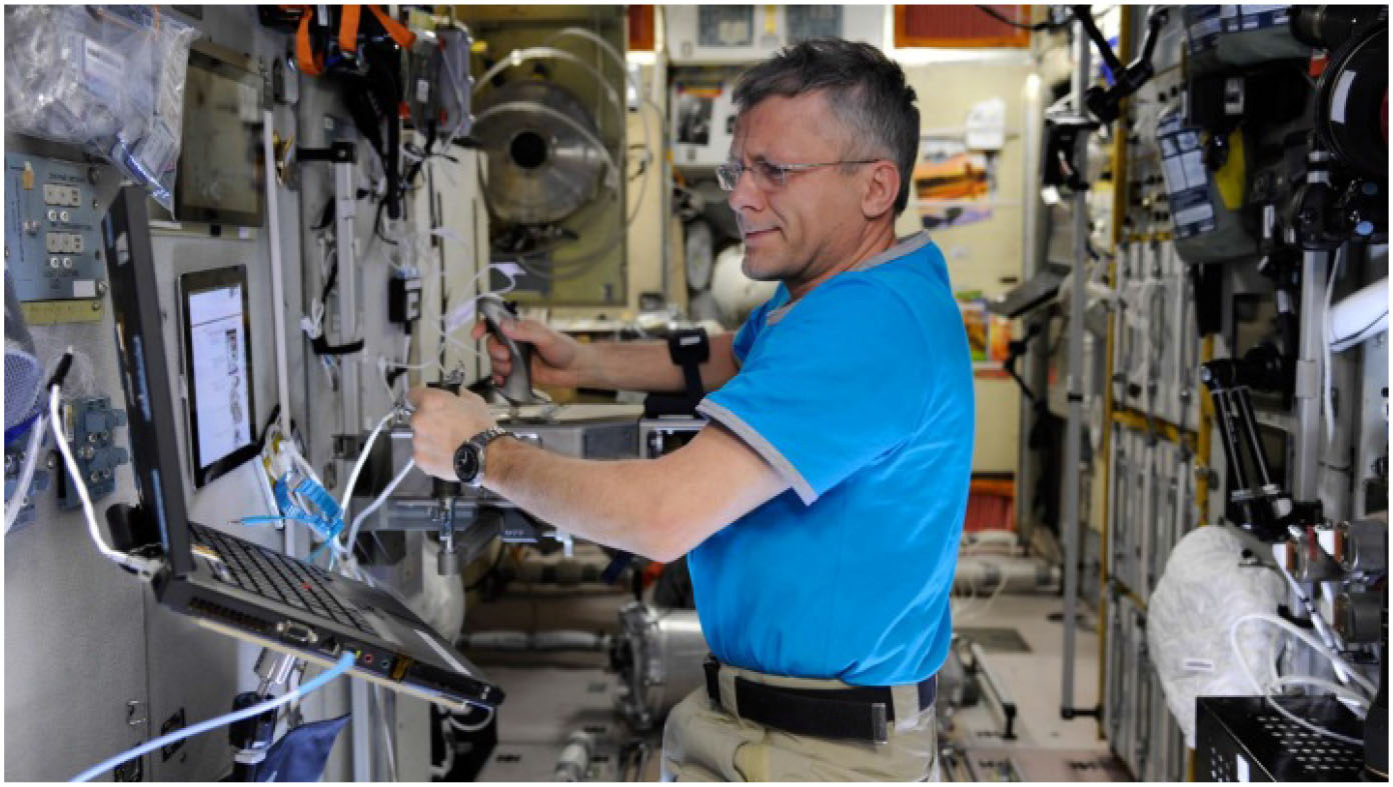
Experimental setup on board the International Space Station. Cosmonaut during the system check.

The same experimental apparatus and task paradigm were used as in the prior studies. In the terrestrial pre- and post-mission sessions, cosmonauts performed the experiment in the Gagarin Cosmonaut Training Center in Moscow in a setup similar to studies 1 and 2. In the mission sessions, the joystick and experimental notebook were installed at a module wall of the Russian Zvezda module of the International Space Station ISS (refer to Figure 3). Body stabilization in weightlessness was achieved by a foot rail, a handle for the left hand and the fixation strap at the joystick's armrest. Although the cosmonauts performed the task aboard the ISS in an upright position, the tasks in the terrestrial sessions were performed in a seated position as in Studies 1 and 2. This decision was related to the fact that the authors found no influence of position (sitting vs. standing) on experimental task performance in preliminary studies and, furthermore, standing performance in the terrestrial condition was perceived as unnecessarily strenuous.

#### 4.1.3. Experimental design, procedure, and schedule

The experimental design and procedure were the same as in Study 2. The experimental schedule of Study 3 has been described above. The complete experiment was conducted in the framework of the Kontur-2 space project (2012–2018).

#### 4.1.4. Data analysis

Due to the small sample size, non-parametric Quade tests (Quade, 1979) and corresponding *post-hoc* comparisons (Conover, 1980) with Benjamini-Hochberg α level adjustment (FDR = 0.1, Benjamini and Hochberg, 1995; cf. Weber et al., 2021) were performed on the same measures as in the previous studies. As time effects were identified for RT, FMT, and Tracking Error in Study 2, the expected ranks for the five sessions in the Quade tests were adjusted accordingly (RT: 5,4,3,2,1; FMT and Tracking Error: 5,4,3,2,2). For RMT, no such time effect was found and the expected rank was chosen for all sessions (3,3,3,3,3).

### 4.2. Results

#### 4.2.1. RT and RMT

No significant effects were found for both measures, neither across all haptic conditions nor for the isotonic condition alone (see Tables 6, 7).

**Table 6:** Session effects on performance measures: Means, SD (in parentheses), and Quade main effects.

	**T1**	**T2**	**T3**	**T4**	**T5**	**Quade**
**Study 3: Session Effects**						
RT [s]	0.546 (0.172)	0.499 (0.176)	0.484 (0.192)	0.460 (0.174)	0.459 (0.160)	n.s.
RMT [s]	1.242 (0.710)	1.216 (0.876)	1.281 (0.997)	1.087 (0.846)	1.089 (0.878)	n.s.
FMT [s]	5.027 (1.213)	5.220 (1.019)	6.102 (1.748)	5.129 (0.450)	4.889 (0.826)	n.s.
Tracking error [mm]	2.288 (0.198)	1.824 (0.200)	1.896 (0.085)	1.673 (0.268)	1.595 (0.305)	n.s.

**Table 7:** Effects of haptic settings on performance measures: Means, SD (in parentheses), and Quade main effects.

	**Isoton**.	**Stiff. 1**	**Stiff. 2**	**Stiff. 3**	**Damp. 1**	**Damp. 2**	**Mass 1**	**Mass 2**	**Quade**
**Study 3: Haptic Setting Effects**									
RT [s]	0.441 (0.135)	0.440 (0.151)	0.540 (0.237)	0.454 (0.163)	0.497 (0.165)	0.503 (0.243)	0.518 (0.125)	0.524 (0.136)	n.s.
RMT [s]	1.156 (0.940)	1.285 (1.011)	1.339 (1.042)	1.324 (0.971)	0.982 (0.527)	1.190 (0.825)	1.109 (0.743)	1.080 (0.760)	n.s.
FMT [s]	5.257 (0.760)	5.546 (0.253)	4.816 (0.305)	4.610 (0.520)	5.887 (1.747)	5.683 (2.415)	5.162 (1.162)	5.228 (1.164)	n.s.
Tracking error [mm]	1.956 (0.074)	1.868 (0.432)	1.865 (0.358)	1.593 (0.296)	1.795 (0.139)	1.906 (0.210)	1.980 (0.261)	1.881 (0.182)	n.s.

#### 4.2.2. Fine motion time

**Figure 4:**
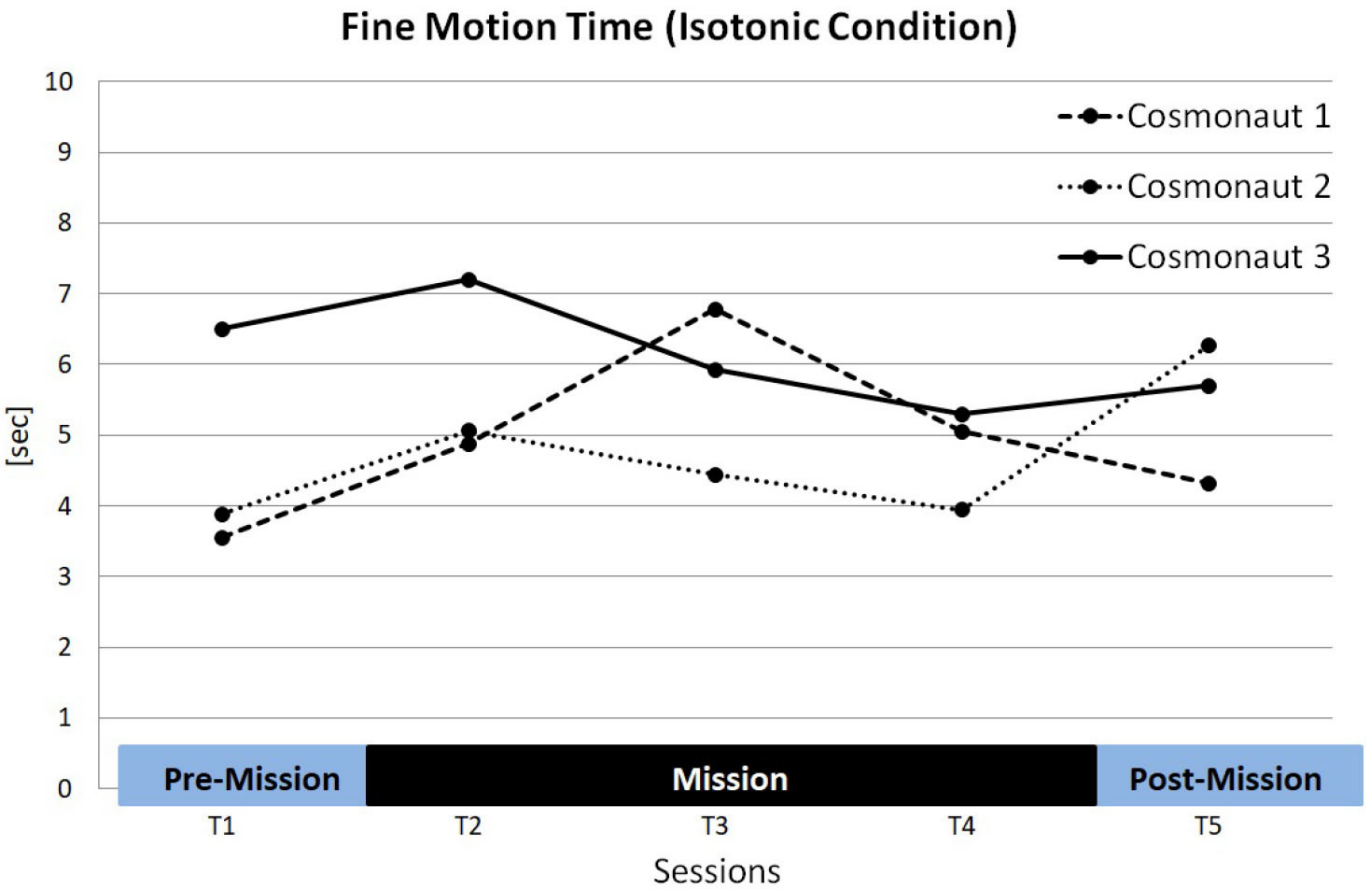
Individual fine motion times (FMT) of the three cosmonauts in the isotonic baseline condition.

No Session effect was evident across all conditions [*F*_(4, 8)_ = 2.60, ns.] as well as for the isotonic condition exclusively [*F*_(4, 8)_ = 2.45, ns.] (see Table 6). Nevertheless, all astronauts showed longer FMTs in T2 compared to T1 (*p* = 0.05 (1tt); Cosm1: +37.5%; Cosm2: +30.1%; Cosm3: +11.0%, see Figure 4). In general, a clear deviation from the expected learning trend was found, i.e., FMTs did not decrease across time and consequently, all *post-hoc* comparisons of T1 and the subsequent sessions reached significance (*p*_(T1vs.T3;T4;T5)_ < 0.05). Cosmonaut 1 not only showed the highest increase of FMTs from T1 to T2, his FMTs even further increased in T3, while the opposite was true for the other two cosmonauts.

**Figure 5:**
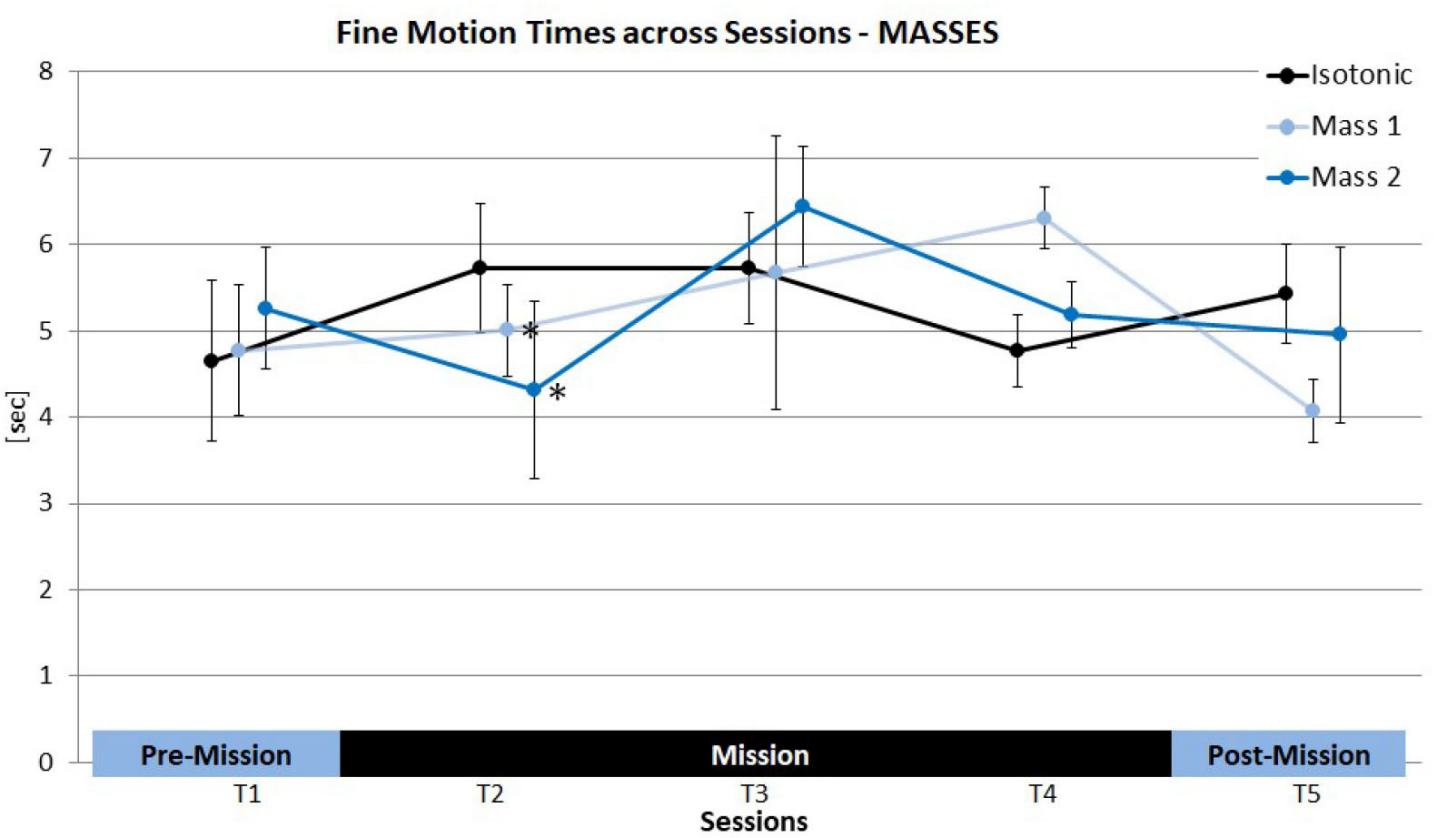
Overview of stiffness effects on fine motion times (FMT) compared to the isotonic baseline condition (black line) across sessions (means ± SE). *p < 0.05.

No significant overall effects of HAPTIC SETTING were evident across sessions and in all individual sessions (see Table 7). However, when solely comparing the two masses with the isotonic baseline in T2, a marginally significant HAPTIC SETTING effect was found [*F*_(2, 4)_ = 5.63, *p* = 0.07]. Compared to the isotonic condition (*M*= 5.7 s) FMTs were shorter with Mass 1 (Cosm1: −16.6%, Cosm2: −5.3%, Cosm3: −19.9%;*M*= 5.0 s, *p* = 0.05; 1tt) and Mass 2 (Cosm1: −4.1%, Cosm2: −113.3%, Cosm3: −22.9%; *M*= 4.3 s, *p* < 0.05; 1tt) conditions (see Figure 5). This overall effect pattern was not evident in the later sessions. Yet, FMTs of Cosmonaut 1 were also improved by virtual mass compared to the isotonic condition in T3 (Mass 1: −10.7%; Mass 2: −8.7%).

**Figure 6:**
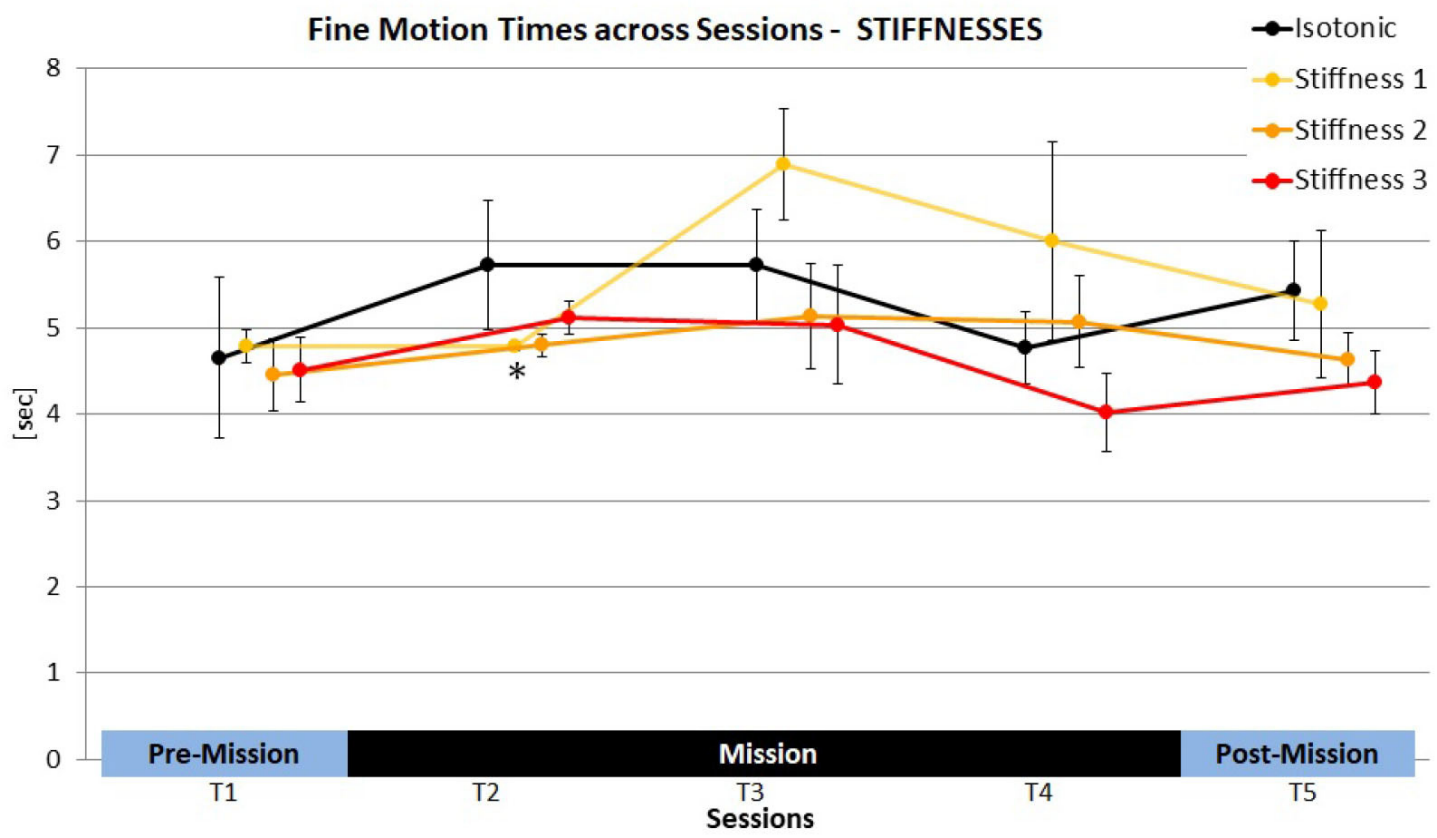
Overview of virtual mass effects on fine motion times (FMT) compared to the isotonic baseline condition (black line) across sessions (means ± SE). *p < 0.05.

Similarly, no significant main effect was found when comparing stiffnesses and the isotonic condition in T2 [*F*_(3, 6)_ = 4.8, ns.]. Still, FMTs in T2 were shorter for Stiffness 1 compared to the isotonic condition (Cosm1: −0.8%, Cosm2: −7.5%, Cosm3: −32.8%;*M*= 4.8 s, *p* < 0.05; 1tt) and the same pattern also emerged when comparing Stiffness 2 and the isotonic condition in T2 (Cosm1: −5.0%, Cosm2: −7.2%, Cosm3: −30.0%; see Figure 6) although the *post-hoc* comparison failed to reach significance in this case (*M*= 4.8 s, *p* = 0.14; 1tt). In T3, FMTs of Cosmonaut 1 still improved with Stiffness 1 (−6.2%), 2 (−27.2%), and 3 (−35.9%) compared to the isotonic condition. For the other cosmonauts, no such benefits of stiffness were evident, since their performance in the isotonic condition clearly improved from T2 to T3. Stiffness 1 even led to increased FMTs compared to the isotonic condition for these subjects (Cosm2: +38.2%, Cosm3: +37.6%).

#### 4.2.3. Tracking error

No significant overall effects on tracking error were found (see Tables 6, 7). However, when analyzing the data for the vertical subtask only, the Quade test yielded a significant HAPTIC SETTING effect [*F*_(7, 14)_ = 3.07, *p* < 0.05], indicating that tracking errors in the isotonic condition were significantly greater compared to the Stiffness 3 (*p* < 0.01) and Damping 1 condition (*p* < 0.05).

### 4.3. Discussion

In Study 3, the effects of microgravity and haptic support on aiming and tracking performance were investigated in a spaceflight mission. It was hypothesized that subtasks with high precision demands should be mainly affected in the initial phase of exposure to microgravity. Indeed, the times for finely matching the aiming targets (FMT) increased for all subjects when performing the task in the initial mission session after 2 weeks of spaceflight. Surprisingly, however, no evidence for any negative effects was found for tracking precision. This is also in contrast to earlier findings with the very same tracking task paradigm, but position control (Weber et al., 2021). Consequently, Hypothesis 1 is only confirmed for the aiming but not for the tracking task. A plausible explanation for the presence of microgravity effects on aiming precision and the absence of such effects for tracking precision are the different proportions of feedforward vs. feedback controlled motions. During precision aiming, the target zone is reached and a series of motion corrections are performed at a very low speed. Here, feedback control is mainly involved, and visual as well as proprioceptive information is processed to a large extent (Desmurget and Grafton, 2000). As described above, the given target speed has to be matched during tracking with velocity control. Here, larger positional errors occur much faster and have to be corrected by rapid feedforward-planned movements—similar to rapid aiming motions. Still, feedback control does play an important role, but to a lesser extent compared to the precision aiming task with velocity control or when tracking is performed with position control.

Aiming accuracy in the first session in microgravity improved for all cosmonauts with both virtual masses (0.00187 and 0.00374 kg·m^2^) and low to medium spring stiffness (0.262 and 0.524 Nm/rad). Additionally, in the second mission session (4 weeks in space) a positive effect of mass and stiffness on fine motion times was also found for the cosmonaut who still showed performance decrements in this phase. For the other two cosmonauts, whose performance level improved again in this session, the low stiffness even proved to be a disadvantage compared to no haptic support. In sum, however, Hypothesis 2 is confirmed for the aiming task. As in previous studies (Weber et al., 2021, 2022), low to moderate stiffness proves to be particularly suitable for reducing the negative effects of microgravity. Surprisingly—and contrary to the findings of the previous studies—we also found a positive effect of virtual mass in the first spaceflight sessions. The general advantage of higher inertia of the input medium (with velocity control) is that unintended velocity changes (e.g., tremor) are avoided. In precision aiming, this is particularly useful when the final target position is to be held exactly. Altogether, the two forms of haptic feedback seem to optimally compensate for the proprioceptive losses in the initial phase of adaptation to microgravity. While the spring stiffness helps to perceive the position of the limbs when approaching a target, the additional mass helps to hold a specific position precisely.

## 5. General discussion

The main objective of the present series of studies was to investigate sensorimotor impairments in microgravity and whether haptic support can be used to maintain performance. Sensorimotor performance was investigated using two classic experimental tasks in this research field: aiming and tracking tasks. In experimental paradigms utilizing joysticks as human-machine interfaces, two different transfer functions have been implemented: zero-order transfer functions (i.e., position control) and first-order transfer functions (i.e., velocity control). In the current studies, the very same experimental paradigm was implemented as in earlier studies of the authors (Weber et al., 2020, 2021, 2022), but instead of position control, velocity control was used. In two terrestrial studies, the impact of cognitive load on task performance and potential time effects and interactions with haptic support were explored. The results of these studies were important for the analysis and interpretation of the concluding spaceflight study, in which three cosmonauts performed the same experimental tasks in different stages of a space mission (2, 4, and 6 weeks in space) and during terrestrial pre- and post-flight sessions. Combining the current and previous findings of the authors, a comprehensive and clearer picture of sensorimotor performance in microgravity conditions emerges and a more detailed analysis of when and why performance impairments occur is possible.

### 5.1. Sensorimotor impairment during spaceflight: The trigger mechanisms

Previous research has discussed several possible trigger mechanisms for sensorimotor impairments in microgravity. There seems to be a key variable to better assess the relevance of the different mechanisms: the temporal pattern when these impairments occur (e.g., Manzey et al., 2000). From a methodological point of view, longitudinal experimental designs are utilized. However, general time effects (e.g., de-motivation, learning) play an important role in such designs and thus complicate the analysis of potential microgravity-related effects. For this reason, the terrestrial study on time effects was carried out and revealed significant learning effects for most performance measures (except for rapid motion times, RMT). Contrary to the expected learning effects, however, the spaceflight study showed that the time needed to precisely hit the target position (i.e., fine motion times, FMT) deteriorated for all cosmonauts in the first session in microgravity (after 2 weeks in space). This trend continued for one cosmonaut even until the 4th week in space. Both the general temporal pattern and the affected performance dimension are similar to the previous findings on aiming performance with position control (Weber et al., 2022). This provides further evidence for the assumption that distorted proprioception is the main trigger underlying this performance decrements.

This interpretation is also further supported by the results of the current study, as well as earlier findings (Weber et al., 2019), on the influence of additional cognitive load induced by a secondary task. Cognitive load did not have any impact on fine motion times neither for position nor for velocity control—but exactly and exclusively these performance dimension was affected by microgravity.

This finding is particularly interesting as the implemented velocity control scheme substantially increases task complexity and hence leads to significantly higher cognitive load compared to position control as reported earlier (Schäffler, 2016). Consequently, attentional deficits due to general mission-related workload should have had a much stronger effect in the present study, but still no evidence was found for such effects.

The tracking task results also complement this picture very well. While the study implementing position control showed that the accuracy of the tracking deteriorated significantly in the earlier phase of the space flight (2 weeks in space), this could not be observed with velocity control in any phase of the space mission. As discussed above, feedback control seemingly plays a less important role during tracking with velocity compared to position control, and thus proprioceptive deficits do not have an impact here. Study 1 revealed higher tracking errors when subjects' cognitive resources are limited. Yet, the absence of any effects in the spaceflight study again provided evidence that attentional deficits were not relevant here. At the same time, these findings shed more light on the preconditions when attentional deficits do have an impact on tracking performance. In the studies reporting tracking performance degradations due to insufficient cognitive resources, an unstable tracking task had to be performed, that is, random target deflections from an ideal position had to be compensated, which of course further increases the task complexity compared to the current stable tracking task (Manzey et al., 1993, 2000; Bock et al., 2003). Therefore, it is conceivable that cognitive demands in the current stable and continuous tracking task were not sufficiently high to find similar effects.

On basis of the described pattern of results, it can also be ruled out that an altered motion strategy had a relevant impact here. Such a strategy should have mainly affected rapid motions, and a potential slowing of motions should have been evident across all phases of exposition to microgravity (Berger et al., 1997; Clément et al., 2020).

When interpreting the findings, it must be mentioned that the presented experiments with velocity control were carried out with the same cosmonauts and on the same mission days as the experiments on position control (Weber et al., 2021, 2022), always following position control. This was done to better compare the effects of microgravity exposure across both control modes. It is however conceivable this chronology could have had an effect on results. For various reasons, however, this does not seem to be the case. (1) Task demands are substantially different for velocity control, as evidenced by learning effects over time (refer to Study 2) that are even more pronounced than for position control. Apparently, there was no significant transfer from one control mode to the other. (2) The effects of microgravity on performance dimensions, which are mainly based on feedback control, are evident in both control modes. Apparently, no significant adaptation occurred within the mission sessions.

### 5.2. Reinforcing sensorimotor performance in microgravity

The terrestrial Study 2 showed that medium to high stiffnesses (0.524–0.786 Nm/rad) improved fine motion times, while high stiffness reduced tracking errors. Overall, this general pattern is also evident in the spaceflight study where these haptic settings led to the shortest fine motion times and smallest tracking error, although significance was not reached in these cases. More interestingly, however, the optimal haptic values in the initial session in microgravity, where fine motion times were increased for all cosmonauts, were low to medium stiffnesses (0.262–0.524 Nm/rad). This is surprisingly consistent with the findings on precision aiming performance with position control (Weber et al., 2022): low stiffness improved fine motion times in the early phase of spaceflight (2 weeks in space). Additionally, the very same low stiffness also proved to be optimal for improving tracking accuracy (position control) in the same mission phase (Weber et al., 2021). Obviously, the positive effects of subtle haptic cues provided by the human-machine interface generalize across different control modes and experimental tasks, although the precise values of spring stiffness have to be adjusted for each control mode and its typical workspace. The finding, that virtual mass also leads to improved fine motion times during the first weeks of exposition to microgravity, however, seems to be related to the specific challenges of velocity control. Precisely holding a target position while any positional inaccuracy transfers into the velocity of the controlled object was the most demanding subtask of this experiment. Here, the higher (virtual) mass of the input device seems to be the optimal support.

The synopsis of these empirical results also allows for an interesting insight into the functional role of haptic feedback in microgravity. Any sensorimotor performance depends on accurate perception of body or limb positions. In microgravity, afferent sensory feedback from the proprioceptive system is distorted (e.g., Lackner and DiZio, 1992). This has been explained by reduced muscle tone in microgravity, which seemingly affects muscle spindle sensitivity (Lackner and DiZio, 2000; Proske, 2019). The above findings suggest that it is less plausible to assume that haptic feedback generally increases the muscle resting tone and thus restores the proprioceptive function. In general, the present results are in line with observations that muscle spindle sensitivity can be restored by a comparatively small counterforce (requiring 10% of the maximum voluntary contraction, refer to Ansems et al., 2006). However, if muscle spindles sensitivity can be re-established by muscle contraction, then this effect should also occur or even be more evident with high stiffness and damping, since the precise approach to the target position requires working against a larger or continuous force, respectively. Yet, we did not find any positive effect of these haptic settings on aiming performance. The fact that spring stiffness is beneficial is more likely to indicate that this haptic feedback of limb positions helps to compensate for the distorted proprioception and serves as an alternative source for the sense of limb position. Here, even low stiffness seems to be sufficient to achieve the desired effect, whereas movements against higher counterforces in weightlessness are often difficult to stabilize and result in worse performance (Weber et al., 2019, 2020, 2021). This is a great advantage as lower spring stiffnesses not only increase movement precision but also do not hinder the execution of fast and dynamic movements. While spring stiffness plays a role when correcting larger deviations that require larger joystick deflections, virtual mass is mainly relevant when trying to exactly match and hold the desired position. In this final homing-in phase of the aiming task, minimal joystick deflections are performed to finely adjust the joystick position. Here, inertia haptically indicates and prevents deviations from the target position. The fact that virtual mass had a positive effect only in the initial mission phase and seems to be more of a hindrance in the later mission phases again provides evidence that impaired proprioception can be compensated for by this form of haptic.

## 6. Limitations

As in many space studies, the small number of cases is a substantial limitation to the interpretation of the results. This is further complicated by the influence of inter-individual differences in the response to microgravity (Kornilova, 1997; Bock, 1998). Similarly, the robustness of recorded data is potentially limited, since no trial repetitions for each target position and tracking direction could be performed due to the very limited experimental time on board the ISS. Nevertheless, a consistent overall finding emerges across the authors' current and previous studies. The small sample size is also a limitation of Study 1, which was essentially conducted to examine whether previous findings on the influence of cognitive load in position control can be transferred to velocity control. Although the results of the earlier and the present study are very similar, the lack of statistical power still is a methodological shortcoming of Study 1. The generalizability of the findings is also restricted in that one specific design of a joystick was implemented and it remains unclear to what extent the results on haptic support can be transferred to other human-machine interfaces with more degrees of freedom, other movement scaling, and hence workspaces.

## 7. Conclusion

In the present series of studies, it has been demonstrated that microgravity impairs the sensorimotor performance of humans, especially in the early phase of adaptation to this environment. This confirms the findings of previous studies and provides further evidence for the assumption that distorted proprioception is the main reason for these performance impairments. The proprioceptive deficit can be compensated by means of haptic feedback provided by the human-machine interface. While this has been shown for position control in previous work, the present study investigating velocity control provides further evidence that virtual mass and moderate degrees of spring stiffness seem to optimally support the limb position sense and thus fine motor performance during microgravity adaptation. From a theoretical perspective, the current study contributes to a better understanding of the main causal mechanisms behind sensorimotor impairments in altered gravity conditions. As a practical implication, the study also provides important hints on how to optimize the human-machine interfaces of telerobotic systems (which are usually position or velocity controlled) for future space missions.

## Data availability statement

The raw data supporting the conclusions of this article will be made available by the authors, without undue reservation.

## Ethics statement

The studies involving human participants were reviewed and approved by ROSCOSMOS. The patients/participants provided their written informed consent to participate in this study. Written informed consent was obtained from the individual(s) for the publication of any potentially identifiable images or data included in this article.

## Author contributions

BW designed and conducted all experiments, performed the statistical analyses, and wrote the first version of the manuscript. MS was responsible for software development and implementation. Both authors contributed to manuscript revision, read, and approved the submitted version.

## Conflict of interest

The authors declare that the research was conducted in the absence of any commercial or financial relationships that could be construed as a potential conflict of interest.

## Publisher's note

All claims expressed in this article are solely those of the authors and do not necessarily represent those of their affiliated organizations, or those of the publisher, the editors and the reviewers. Any product that may be evaluated in this article, or claim that may be made by its manufacturer, is not guaranteed or endorsed by the publisher.

## Abbreviations

HMI: Human-Machine Interface
GUI: Graphical User Interface
RT: Reaction Time
RMT: Rapid Motion Time
FMT: Fine Motion Time
rmANOVA: repeated measures Analysis of Variance.
